# Ultrasound-Based Biofeedback for Pelvic Floor Rehabilitation: A Systematic Review of Randomized Controlled Trials

**DOI:** 10.3390/jcm15166422

**Published:** 2026-08-19

**Authors:** César Garrido-Fernández, Eva Lantarón-Caeiro, Isabel Escobio-Prieto, Pablo Hernandez-Lucas

**Affiliations:** 1 Faculty of Physiotherapy, University of Vigo, Campus A Xunqueira, 36005 Pontevedra, Spain; cesarantonio998@gmail.com (C.G.-F.); evalantaron@uvigo.es (E.L.-C.); phernandez@uvigo.es (P.H.-L.); 2 Clinical Physiotherapy Research Group (FS1), South Galicia Health Research Institute (IIS South Galicia), SERGAS-UVIGO, 36005 Pontevedra, Spain; 3 Departamento de Fisioterapia, Instituto de Biomedicina de Sevilla (IBIS), Universidad de Sevilla, 41902 Seville, Spain; 4 Research Group CTS-1174, Human Function Research Group, FunctionLab, 41008 Seville, Spain; 5 Research Group Hi10, Universidade de Vigo, Campus A Xunqueira, 36005 Pontevedra, Spain

**Keywords:** physiotherapy, pelvic floor, ultrasonography, biofeedback, urinary incontinence, pelvic pain

## Abstract

Pelvic floor dysfunctions are highly prevalent and can substantially impair quality of life. Physiotherapy is considered a first-line conservative treatment, and the integration of technologies such as ultrasound may enhance outcomes through visual biofeedback. **Objectives**: To evaluate the effectiveness of ultrasound as a biofeedback tool in physiotherapeutic interventions for pelvic floor dysfunctions across different populations. **Methods**: A systematic review was conducted following PRISMA guidelines and registered in PROSPERO (CRD420261278982). The literature search was conducted between February and March 2025 in PubMed, Web of Science, Scopus, Medline, and PEDro. Randomized controlled trials using ultrasound as biofeedback in physiotherapy interventions were included. Methodological quality was assessed using the PEDro scale, and risk of bias was evaluated with the RoB 2 tool. **Results**: Two randomized controlled trials involving 75 women were included. One study investigated older women with urinary incontinence, whereas the other included postpartum women with pregnancy-related pelvic girdle pain. In Galea et al., transabdominal ultrasound biofeedback was not superior to conventional feedback based on vaginal palpation for urinary leakage, incontinence frequency, or quality of life, although the ultrasound group showed a significant within-group reduction in leakage episodes. In Kuo et al., ultrasound-guided biofeedback combined with pelvic floor and stabilization exercises reduced pain and disability compared with education alone and improved walking performance, but it was not consistently superior to exercise without biofeedback and did not significantly improve pelvic floor muscle contractility. The small number of studies, heterogeneity of populations and interventions, and low to very low certainty of the evidence limited the strength of the findings. **Conclusions**: Ultrasound-based biofeedback may be a useful adjunct to pelvic floor physiotherapy, particularly for facilitating correct muscle contraction and supporting motor learning. However, the available evidence does not demonstrate a consistent additional clinical benefit over conventional feedback or exercise alone. These findings should therefore be interpreted cautiously. Further high-quality randomized controlled trials with larger samples, standardized protocols, and longer follow-up are required.

## 1. Introduction

Pelvic floor dysfunction (PFD) constitutes a highly prevalent public health condition worldwide, with a substantial impact on patients’ quality of life, daily functioning, and psychosocial well-being [1]. In women, the prevalence is estimated at approximately 28.8%, being strongly associated with factors such as age, parity, and body mass index [2]. The most common manifestations include urinary incontinence, pelvic pain, pelvic organ prolapse, and fecal incontinence [3]. In men, although prevalence is lower and often underdiagnosed due to less evident symptomatology, conditions such as post-prostatectomy urinary incontinence, chronic pelvic pain syndrome/chronic prostatitis, and erectile dysfunction are frequently reported [4]. In the pediatric population, although prevalence is lower, PFD is often linked to functional disorders such as constipation, with clinical presentations including bladder–bowel dysfunction, vesicosphincteric dyssynergia, and nocturnal enuresis [5,6]. Across all populations, these disorders significantly impair physical, emotional, and social health, highlighting the need for effective and accessible therapeutic strategies [2].

Pelvic floor physiotherapy is currently considered a first-line treatment for PFD [7]. Core interventions include pelvic floor muscle training (PFMT) [8], manual therapy techniques [9], and electrotherapy, which can in turn be combined with PFMT approaches [10]. In this context, biofeedback (BF), both visual and auditory, has been widely used to improve motor awareness, coordination, and quality of muscle contraction, with evidence suggesting that its integration with PFMT may accelerate recovery and optimize clinical outcomes [11].

In recent years, technological advances have led to increasing interest in the use of ultrasound in physiotherapy, particularly within pelvic floor rehabilitation [12]. Beyond its diagnostic role, rehabilitative ultrasound imaging (RUSI) enables real-time visualization of muscle activity, which may facilitate motor learning and improve patients’ ability to correctly activate pelvic floor musculature during exercise [13]. Additionally, advanced modalities such as three-dimensional ultrasound allow more precise and dynamic assessment of pelvic floor structures, potentially enhancing treatment monitoring and long-term outcomes [14].

Current evidence and major clinical guidelines such as “Urinary incontinence and pelvic organ prolapse in women: management” [15] and “EAU Guidelines on Urinary Incontinence” [16] support the use of physiotherapy and biofeedback-based interventions as evidence-based approaches for managing PFD. Previous systematic reviews have examined the role of RUSI in assessing muscle morphology and motor control [17], as well as its diagnostic value in pelvic organ prolapse [18]. However, these reviews have mainly focused on ultrasound as an assessment or diagnostic tool rather than as an active therapeutic biofeedback component. In contrast, the present review specifically examines the clinical effectiveness of ultrasound as a visual biofeedback tool within pelvic floor physiotherapy interventions across different patient populations and clinical contexts. This distinction is clinically relevant because visual feedback may facilitate motor learning by improving patients’ awareness of pelvic floor muscle contraction, allowing real-time correction of incorrect activation patterns, and reinforcing appropriate sensorimotor strategies during exercise.

Therefore, the aim of this systematic review is to synthesize and critically analyze the available evidence regarding the effectiveness of ultrasound as a visual biofeedback tool in physiotherapy interventions targeting pelvic floor dysfunctions across diverse populations.

## 2. Materials and Methods

### 2.1. Design

This systematic review was conducted and reported in accordance with the Preferred Reporting Items for Systematic Reviews and Meta-Analyses (PRISMA 2020) statement [19] and the recommendations of the Cochrane Collaboration [20]. The completed PRISMA 2020 Checklist is provided as Supplementary Material. The review protocol was registered in PROSPERO (CRD420261278982). Searches were conducted in February and March 2025 across five electronic databases: PubMed, Web of Science, Medline, Scopus, and PEDro. The search equation included a combination of MeSH terms (*Ultrasonography*, *Exercise Therapy*, *Physical Therapy Modalities*, *Feedback*, *Sensory*, *Pelvic Floor*, *Pelvic Floor Disorders*, *Vaginismus*, *Uterine Prolapse*, *Dyspareunia*, *Urogenital Diseases*) and free terms to broaden the search strategy: (*Ultrasound*, *Echography*, *Sonography*, *Motor control*, *Rehabilitation Exercise*, *Physiotherapy*, *Rehabilitation*, *Biofeedback*, *Visual Feedback*, *Sensorimotor*).

The search strategy was developed based on the PICOS framework: Population (patients with pelvic floor dysfunctions), Intervention (physiotherapy interventions using ultrasound as biofeedback or sensory feedback), Comparison (interventions without ultrasound or no intervention), Outcomes (improvements in symptoms or pelvic floor function), and Study design (randomized controlled trials). Language restrictions were applied, and only studies published in English or Spanish were considered eligible. No restrictions regarding publication year were applied. All records available from database inception to the final search date were considered eligible for screening.

### 2.2. Study Selection

Following the removal of duplicates, titles and abstracts were independently screened by two reviewers to identify potentially eligible studies. Inter-reviewer agreement was assessed using Cohen’s kappa coefficient, showing almost perfect agreement (κ = 0.98) [21]. Subsequently, full-text articles were retrieved and evaluated for eligibility based on the predefined inclusion and exclusion criteria. Disagreements between reviewers were resolved by discussion, and when necessary, by consultation with a third reviewer until consensus was reached.

The inclusion criteria were as follows: (I) studies involving individuals with pelvic floor dysfunctions; (II) randomized controlled trials; and (III) interventions incorporating ultrasound as a biofeedback tool. Exclusion criteria included: (I) studies focused on prevention rather than treatment of pelvic floor dysfunctions; and (II) studies addressing conditions outside the pelvic floor complex.

### 2.3. Data Extraction

Data extraction was performed independently by two reviewers (C.G.-F., P.H.-L.) using a standardized data extraction form. Any discrepancies were resolved through discussion, and when necessary, by consultation with a third reviewer (E.L.-C.) to reach consensus.

The following data were extracted from each included study: (I) general study information (authors, year of publication, and journal); (II) participant characteristics (sample size, age, sex, and inclusion/exclusion criteria); (III) intervention details (type, duration, frequency, and specific use of ultrasound as biofeedback); and (IV) outcome measures (variables assessed, measurement instruments, and follow-up periods).

When necessary, corresponding authors were contacted to obtain missing or unclear data. Extracted data were synthesized and presented in structured tables to facilitate comparison across studies (Table 1).

### 2.4. Quality Assessment

The methodological quality of the included studies was assessed using the Physiotherapy Evidence Database (PEDro) scale [22]. This scale evaluates key aspects of methodological quality in clinical trials, including eligibility criteria, random allocation, allocation concealment, baseline comparability, blinding, follow-up, intention-to-treat analysis, between-group comparisons, and reporting of point estimates and variability. The risk of bias was evaluated using the Cochrane Risk of Bias 2.0 (RoB 2) tool [20], considering the following domains: randomization process, deviations from intended interventions, missing outcome data, measurement of the outcome, and selection of the reported result. In addition, the certainty of the evidence was assessed using the Grading of Recommendations Assessment, Development and Evaluation (GRADE) approach [23], taking into account risk of bias, inconsistency, indirectness, imprecision, and publication bias. All assessments were performed independently by two reviewers. Any disagreements were resolved through discussion or by consulting a third reviewer until consensus was reached.

## 3. Results

**Figure 1:**
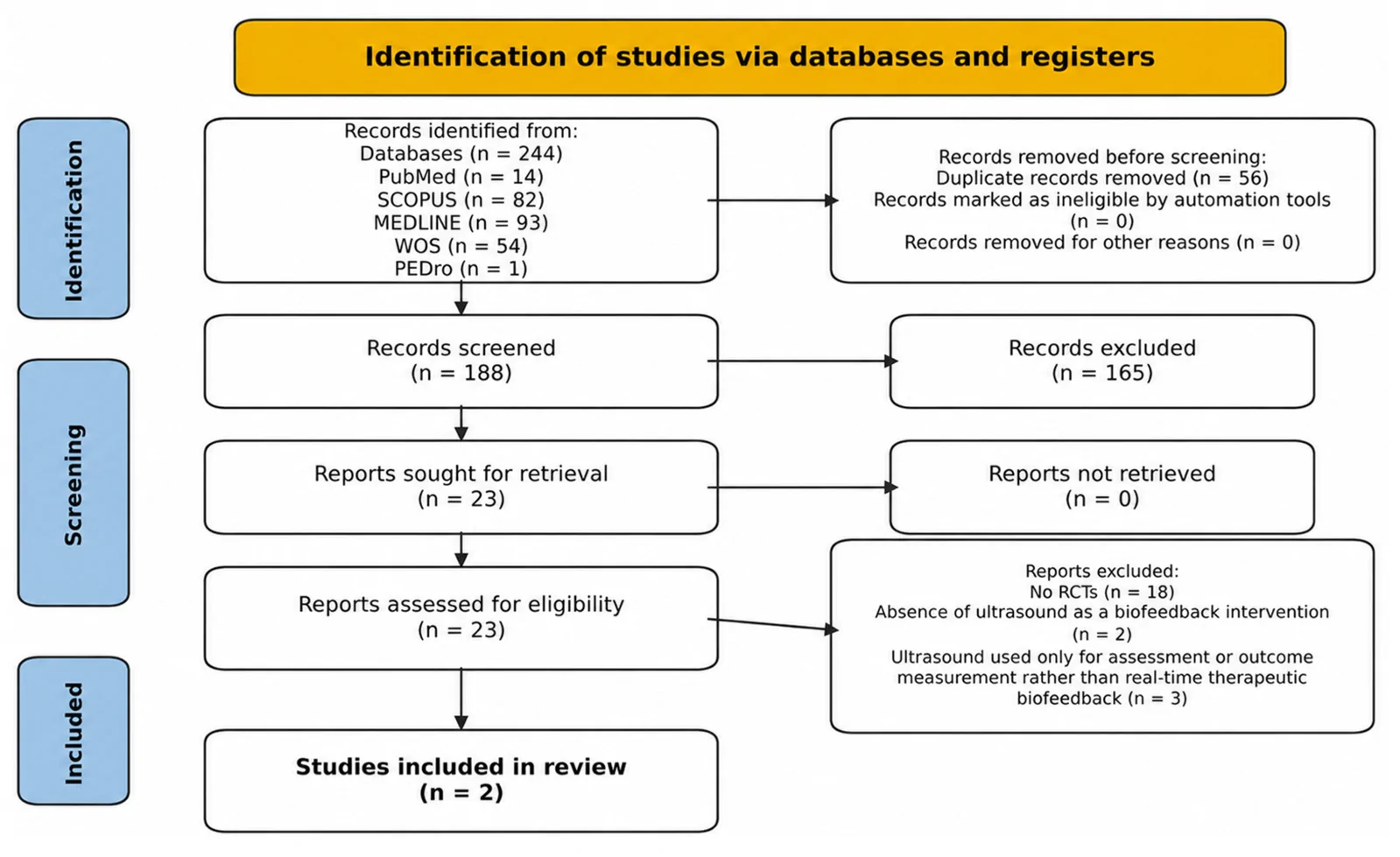
PRISMA 2020 flow diagram of the study selection process. Flow diagram illustrating the identification, screening, eligibility assessment, and inclusion of studies in accordance with the PRISMA 2020 Statement [20].

A total of 244 records were identified through database searching. After the removal of duplicates, 188 studies remained for screening. Following title and abstract screening, 165 studies were excluded. A total of 23 full-text articles were assessed for eligibility. Of these, 23 were excluded: 18 due to not being randomized controlled trials, 2 due to the absence of ultrasound as a biofeedback intervention, and 3 because ultrasound was used only for assessment or outcome measurement rather than as real-time therapeutic biofeedback. Finally, 2 randomized controlled trials [24,25] were included in this systematic review (Figure 1).

### 3.1. Methodological Quality and Risk of Bias of Studies

The methodological quality of the included studies, assessed using the PEDro scale, ranged from 6 to 7 points, indicating moderate-to-high quality (Table 2) [22]. The mean score was 6.5 points. The most common methodological limitations were the lack of blinding of participants and therapists, which was absent in all studies, as well as insufficient allocation concealment in some cases.

Regarding risk of bias, assessed using the RoB 2 tool, all studies were judged to have an overall high risk of bias, mainly due to concerns in the domain of selective outcome reporting (Table 3). The randomization process was considered adequate across all studies, indicating a low risk of bias in this domain.

Both included studies [24,25], were considered to have an overall high risk of bias. In Galea et al. (2013) [24], the main concerns were related to deviations from the intended interventions and selective outcome reporting, whereas Kuo et al. (2024) [25] presented additional methodological limitations regarding allocation concealment, blinding, and outcome reporting, which may reduce the reliability of the findings.

According to the GRADE approach, the certainty of evidence across the included studies was generally low to very low, mainly due to methodological heterogeneity, small sample sizes, and the overall high risk of bias. A summary of the certainty of evidence assessment using the GRADE approach is presented in Supplementary Table S1.

### 3.2. Sample Characteristics

Participant age across the included studies corresponded to adult female populations [24,25]. Galea et al. (2013) [24] included older women with urinary incontinence, whereas Kuo et al. (2024) [25] investigated women with pregnancy-related pelvic girdle pain. The clinical conditions assessed therefore involved two distinct contexts: urinary continence dysfunction in older women and pregnancy-related pelvic pain and functional limitations in women. Both studies also evaluated outcomes related to pelvic floor muscle activation and neuromuscular control [24,25].

### 3.3. Characteristics of the Interventions

Interventions varied between the two included studies [24,25]. Galea et al. (2013) implemented a 10-week pelvic floor muscle training programme in older women with urinary incontinence, using transabdominal ultrasound as visual biofeedback during supervised physiotherapy sessions [24]. Kuo et al. (2024) applied a 4-week ultrasound biofeedback phase followed by progressive lumbopelvic stabilization exercises, with weekly supervised sessions and independent home practice [25].

Regarding intervention content, Galea et al. (2013) compared pelvic floor muscle training supported by transabdominal ultrasound biofeedback with conventional physiotherapy using vaginal palpation to provide feedback on pelvic floor muscle contraction [24]. Kuo et al. (2024) compared a lumbopelvic stabilization programme combined with pelvic floor muscle exercises and ultrasound biofeedback with the same exercise programme without biofeedback [25].

The control conditions also differed between studies. In Galea et al. (2013), the comparison group received conventional pelvic floor muscle training with feedback provided through vaginal palpation [24]. Kuo et al. (2024) included both an active exercise group without ultrasound biofeedback and a control group that received a single educational session on pelvic pain without further exercise intervention [25] (Table 4).

### 3.4. Results of Analyzed Variables

The included studies assessed clinical outcomes related to pelvic floor dysfunctions in two different female populations. Galea et al. [24] evaluated urinary leakage using the 24-h pad weight test, the number of urinary incontinence episodes per week, and quality of life. Kuo et al. [25] assessed pelvic pain, disability, functional performance, abdominal and pelvic floor muscle activation, and bladder displacement.

In older women with urinary incontinence, Galea et al. [24] compared pelvic floor muscle training supported by transabdominal ultrasound biofeedback with conventional physiotherapy using vaginal palpation. No statistically significant between-group differences were observed in the 24-h pad weight test, number of urinary incontinence episodes, or quality of life. However, the ultrasound biofeedback group showed a significant within-group reduction in the number of weekly incontinence episodes. These findings suggest that transabdominal ultrasound may facilitate pelvic floor muscle training, although it did not demonstrate superiority over conventional feedback.

In women with pregnancy-related pelvic girdle pain, Kuo et al. [25] evaluated ultrasound biofeedback combined with pelvic floor muscle training and lumbopelvic stabilization exercises. The ultrasound biofeedback group showed improvements in pelvic pain, muscle fatigue, pelvic floor muscle activation, and bladder displacement. Both exercise groups improved in pain and functional outcomes compared with the education-only control group. However, between-group differences between the ultrasound biofeedback and non-biofeedback exercise groups were not statistically significant for all outcomes.

## 4. Discussion

The aim of this systematic review was to evaluate the effectiveness of ultrasound as a biofeedback tool in the physiotherapeutic management of pelvic floor dysfunctions. Overall, the findings indicate that ultrasound-based biofeedback may support symptom improvement and functional recovery in selected populations; however, its additional benefit over conventional feedback or exercise alone remains uncertain. In older women with urinary incontinence, transabdominal ultrasound was not superior to vaginal palpation for urinary leakage, incontinence frequency, or quality of life. In postpartum women with pregnancy-related pelvic girdle pain, ultrasound-guided biofeedback combined with pelvic floor and stabilization exercises improved pain, disability, and walking performance compared with education alone, although it was not consistently superior to the same exercise programme without biofeedback and did not significantly improve pelvic floor muscle contractility.

The specific contribution of ultrasound-based biofeedback should therefore be interpreted with caution. In both included trials, ultrasound was incorporated within broader physiotherapy programmes that also involved exercise, therapist guidance, education, and repeated clinical contact. Consequently, the observed improvements cannot be attributed exclusively to the ultrasound component and may instead reflect the combined effects of supervised training, treatment intensity, individualized instruction, and patient engagement. This is particularly relevant when comparator groups differed not only in the use of ultrasound but also in the type and amount of feedback provided.

Therapist expertise and technical factors may also influence the effectiveness and reproducibility of ultrasound-based interventions. Its clinical use requires adequate image acquisition, accurate interpretation of pelvic floor movement, and the ability to translate visual information into meaningful real-time instructions. Differences in ultrasound equipment, imaging approach, probe placement, patient position, feedback dosage, session frequency, and progression criteria may therefore contribute to variability between studies. The absence of standardized protocols limits comparability and makes it difficult to identify which intervention components are responsible for the observed clinical effects.

Improved pelvic floor muscle activation and neuromuscular control were relevant outcomes assessed in the included studies [24,25]. Real-time visual feedback provided by ultrasound may facilitate motor learning and enhance the patient’s ability to correctly activate the pelvic floor musculature. Similar effects have been described in other anatomical regions, such as the lumbar [26] and abdominal muscles, where ultrasound has been used to support motor control training [27].

In relation to urinary incontinence, one included study found no significant between-group differences in urinary leakage, incontinence episodes, or quality of life, although the ultrasound group showed a significant within-group reduction in leakage episodes [24]. This is consistent with previous evidence indicating that improved pelvic floor muscle function is associated with better continence control [28]. However, the limited number of included studies prevents firm conclusions regarding the additional benefit of ultrasound-based biofeedback over conventional pelvic floor training [29]. On the other hand, the use of other biofeedback modalities, such as electromyography or intracavitary probes, has also been associated with improvements in pelvic floor dysfunctions [30]. However, several studies have highlighted methodological limitations in their application, including issues related to standardization and patient tolerance [31,32].

The variability in the findings may be related to differences in patient characteristics, intervention protocols, therapist experience, and the specific role assigned to ultrasound within the rehabilitation program. For instance, previous research has reported that although correct muscle contraction can be visualized using ultrasound, no significant differences were observed in treatment efficacy between ultrasound-guided interventions and standard approaches [29]. Therefore, the effects of ultrasound as a biofeedback tool may not be consistent across all pelvic floor conditions, and its additional value may depend on whether ultrasound is used as a central therapeutic feedback component or only as an adjunct within support within a broader rehabilitation program.

Patient adherence is another relevant factor. Ultrasound as a biofeedback tool provides immediate visual information, which may enhance patient engagement during treatment. Previous research has highlighted the potential role of visual feedback in improving adherence and motor learning during rehabilitation. However, adherence is likely to depend not only on the biofeedback modality itself, but also on therapist interaction, supervision, treatment frequency, and follow-up [31].

Another potential advantage of ultrasound is its safety and tolerability [33]. No adverse events related to ultrasound biofeedback were reported in Galea et al. [24], while Kuo et al. [25] did not identify relevant safety concerns associated with its use. Previous literature supports its safety profile as a non-invasive biofeedback modality [17]. In contrast, other biofeedback approaches, such as endovaginal or rectal probes, have been associated with mild adverse effects, including discomfort during insertion or increased vaginal discharge [34]. These differences may influence patient preference and acceptability in clinical practice [25,34,35].

In Galea et al. [24], transabdominal ultrasound was used primarily as a visual feedback tool to facilitate the learning of correct pelvic floor muscle contraction. However, the study did not demonstrate a clear clinical advantage over conventional feedback based on vaginal palpation, as no significant between-group differences were observed in urinary leakage, incontinence frequency, or quality of life. This finding is consistent with recent evidence indicating that adding feedback or biofeedback to pelvic floor muscle training results in little to no difference in incontinence-related quality of life or patient-reported cure or improvement, and may produce only a small, clinically unimportant reduction in leakage episodes. Therefore, ultrasound may be useful as a non-invasive alternative for teaching pelvic floor muscle contraction, particularly when vaginal palpation is not appropriate or acceptable, but its additional therapeutic benefit remains uncertain [34].

Several limitations should be considered in this review. First, only two randomized controlled trials met the strict eligibility criterion of using ultrasound as real-time therapeutic biofeedback during the intervention, which limits the strength and generalizability of the findings. Second, the protocols were heterogeneous in terms of populations, intervention content, session frequency, ultrasound procedures, comparators, and outcomes. This heterogeneity precluded quantitative synthesis; therefore, a meta-analysis could not be performed. Third, formal assessment of publication bias was not feasible due to the limited number of included studies. In addition, all included studies presented an overall high risk of bias according to the RoB 2 assessment. According to the GRADE approach, the certainty of the available evidence was generally low to very low, which further limits the strength of the conclusions that can be drawn from the current literature. Finally, because ultrasound biofeedback was delivered as part of multimodal physiotherapy programs, it was not possible to isolate its specific effect from other factors such as therapist supervision, education, exercise progression, or treatment intensity. Therefore, the findings of this review should be interpreted with caution.

Future research should include well-designed randomized controlled trials with standardized protocols and longer follow-up periods. Comparative studies evaluating ultrasound against other biofeedback modalities are needed to clarify its relative effectiveness and clinical applicability [31,32]. Furthermore, the integration of ultrasound with digital health strategies, including mobile applications and tele-rehabilitation, represents a promising area for improving accessibility and continuity of care [32,35]. Finally, the evaluation of cost-effectiveness will be essential to support its implementation in clinical settings. Previous evidence highlights the need to consider both clinical outcomes and economic impact when selecting biofeedback strategies, as emphasized by Herderschee et al. (2011) [31].

## 5. Conclusions

Ultrasound-based biofeedback may represent a useful adjunct in the physiotherapeutic management of pelvic floor dysfunctions, particularly for supporting correct muscle activation, neuromuscular control, and improvements in symptoms such as urinary incontinence and pelvic pain in the specific populations analyzed. Real-time visual feedback may enhance motor learning and patient engagement during rehabilitation. However, these findings should be interpreted with caution, given the low to very low certainty of evidence and the high risk of bias identified across the included studies, and the limited number of randomized controlled trials available.

Nevertheless, the current evidence remains preliminary and limited by the small number of randomized controlled trials, heterogeneous intervention protocols, and short follow-up periods. Further high-quality studies with standardized methodologies, larger sample sizes, and longer follow-up are required before ultrasound-based biofeedback can be routinely recommended in clinical practice.

## Figures and Tables

**Table 1:** Search equations.

Databases	Search Equations
**Pubmed**	((“Ultrasonography” [Mesh] OR “Ultrasound” OR “Echography” OR “Sonography”) AND (“Exercise Therapy” [Mesh] OR “Physical Therapy Modalities” [Mesh] OR “Motor control” OR “Rehabilitation Exercise” OR “Physiotherapy” OR “Rehabilitation”) AND (“Feedback, Sensory” [Mesh] OR “Biofeedback” OR “Visual Feedback” OR “Sensorimotor Feedback”) AND (“Pelvic Floor” [Mesh] OR “Pelvic Floor Disorders” [Mesh] OR “Vaginismus” [Mesh] OR “Uterine Prolapse” [Mesh] OR “Dyspareunia” [Mesh] OR “Urogenital Diseases” [Mesh])
**Scopus**	TITLE-ABS-KEY (“ultrasonography” OR “ultrasound” OR “echography” OR “sonography”) AND TITLE-ABS-KEY (“exercise therapy” OR “physical therapy modalities” OR “motor control” OR “rehabilitation exercise” OR “physiotherapy” OR “rehabilitation”) AND TITLE-ABS-KEY (“sensory feedback” OR “biofeedback” OR “visual feedback” OR “sensorimotor feedback”) AND TITLE-ABS-KEY (“pelvic floor” OR “pelvic floor disorders” OR “vaginismus” OR “uterine prolapse” OR “dyspareunia” OR “urogenital diseases”)
**Medline**	((MH “Ultrasonography” OR TX “ultrasound” OR TX “echography” OR TX “sonography”) AND (MH “Exercise Therapy” OR MH “Physical Therapy Modalities” OR TX “motor control” OR TX “rehabilitation exercise” OR TX “physiotherapy” OR TX “rehabilitation”) AND (MH “Feedback, Sensory” OR TX “biofeedback” OR TX “visual feedback” OR TX “sensorimotor feedback”) AND (MH “Pelvic Floor” OR MH “Pelvic Floor Disorders” OR MH “Vaginismus” OR MH “Uterine Prolapse” OR MH “Dyspareunia” OR MH “Urogenital Diseases”))
**WOS**	TS= ((ultrasonography* OR ultrasound* OR echograph* OR sonograph*) AND (“exercise therapy” OR “physical therapy modalities” OR “motor control” OR “rehabilitation exercise*” OR physiotherapy* OR rehabilitation) AND (“sensory feedback” OR biofeedback OR “visual feedback” OR “sensorimotor feedback”) AND (“pelvic floor” OR “pelvic floor disorder*” OR vaginismus OR “uterine prolapse” OR dyspareunia OR “urogenital disease*”))
**PEDro**	“Perineum or genito-urinary system” (body part) and “continence and women’s health” (subdiscipline) Ultrasound AND Visual feedback AND Pelvic floor

**Table 2:** Methodological Quality According to the PEDro Scale.

Author	1 *	2	3	4	5	6	7	8	9	10	11	Score
Galea et al. (2013) [24]	+	+	+	+	−	−	+	+	−	+	+	7
Kuo et al. (2024) [25]	+	+	+	+	−	−	−	+	−	+	+	6

(1) Eligibility criteria were clearly defined; (2) participants were randomly allocated to groups; (3) allocation was concealed; (4) the groups were similar at baseline; (5) all participants were blinded; (6) all therapists were blinded; (7) all assessors were blinded; (8) data were collected from more than 85% of participants; (9) intention-to-treat analysis was performed; (10) statistical comparisons between groups were reported for at least one outcome; (11) both point estimates and measures of variability were provided. * Pertains to external validity and does not contribute to the total score; +: yes; −: no.

**Table 3:** Risk of Bias Assessment Using the RoB 2 Tool.

Author	D1	D2	D3	D4	D5	Overall Risk of Bias
Galea et al. (2013) [24]	+	!	+	+	!	High risk
Kuo et al. (2024) [25]	!	!	+	!	−	High risk

D1: randomization process; D2: deviations from intended interventions; D3: missing outcome data; D4: measurement of the outcome; D5: selection of the reported result; −: high risk; +: low risk; !: some concerns.

**Table 4:** Characteristics of the analyzed studies.

Authors	Sample (% Female)	Groups	Intervention Frequency	Variables Assessed	Results of the Study
Galea et al. [24]	22 (0%)	G1: Standard care/vaginal palpation feedback (*n* = 11). G2: Transabdominal ultrasound visual biofeedback (*n* = 12 randomized; *n* = 11 analyzed).	G1: 10-week PFMT with feedback through vaginal palpation. G2: the same programme using transabdominal ultrasound as real-time visual biofeedback.	Urinary leakage assessed using the 24-h pad weight test; number of urinary incontinence episodes per week; and quality of life.	At post-intervention, median pad weight was 0.9 g (IQR 12) in the standard-care group and 4.8 g (IQR 47.3) in the ultrasound visual-feedback group, with no significant between-group difference (*p* = 0.509). Median weekly leakage episodes were 2 (IQR 7) and 2 (IQR 4), respectively (*p* = 0.664). At 3-month follow-up, pad weight was 0.2 g (IQR 4.2) versus 0 g (IQR 4.8; *p* = 0.428), and leakage episodes were 2 (IQR 3) versus 3 (IQR 5; *p* = 0.868). Only the ultrasound group showed a significant within-group reduction in leakage episodes from baseline to follow-up (*p* = 0.002). No between-group differences were found in quality of life. Effect estimates and 95% CIs were not reported.
Kuo et al., (2024) [25]	53 (100%)	G1 (*n* = 18): LPSE + US-BF-PFMT G2 (*n* = 18): LPSE without BF CG (*n* = 17): education on pelvic pain, without exercises.	G1: 4 weeks with active BF. From week 5: progressive LPSE in G1 and G2. Weekly supervised sessions + independent work.	Pain (NRS), Disability (PGQ), Functional tests: ASLR, TUG, 6 MW, Muscle contractility: thickness of abdominal muscles (TrA, IO, EO) and bladder displacement (PFM), Assessments before and after intervention.	At 8 weeks, BIO + EXE reduced pain compared with CON (1.8 [1.5] vs. 4.4 [1.5]; MD −2.6, 95% CI −3.9 to −1.2; d = 1.737) and disability (14% [10] vs. 28% [21]; MD −14, 95% CI −25 to −2; d = 0.753). EXE also reduced pain compared with CON (2.7 [2.0] vs. 4.4 [1.5]; MD −1.7, 95% CI −3.1 to −0.4; d = 0.976). BIO + EXE improved 6-m walking time compared with CON and EXE, but no significant between-group differences were found for TUG, ASLR fatigue, abdominal muscle thickness, or PFM contractility. Bladder displacement showed a borderline difference versus CON (MD 5.9 mm, 95% CI 0.0 to 11.9; *p* = 0.05).

6 MW: six-minute walk test; ASLR: active straight leg raise; BF: biofeedback; CF-PFMT: conventional pelvic floor muscle training; CG: control group; EO: external oblique; EQ-5D-5L: EuroQol 5-Dimension 5-Level questionnaire; G1: group 1; G2: group 2; ICS-Male SF: International Continence Society Male Short Form questionnaire; IO: internal oblique; IUT: individualized urethral training; LPSE: lumbopelvic stabilization exercises; NRS: numerical rating scale; PFM: pelvic floor muscle; PGQ: Pelvic Girdle Questionnaire; SF-12: 12-item Short Form Health Survey; TrA: transversus abdominis; TUG: timed up and go; US-BF-PFMT: ultrasound biofeedback pelvic floor muscle training.

## Data Availability

The data supporting the findings of this study are available from the corresponding author upon reasonable request.

## Supplementary Materials

The following supporting information can be downloaded at: https://www.mdpi.com/article/10.3390/jcm15166422/s1, Prisma 2020 Checklist; Supplementary Table S1 presents the certainty of evidence assessment according to the GRADE approach.

## Abbreviations

The following abbreviations are used in this manuscript:

BF	Biofeedback
PFD	Pelvic floor dysfunction
PFMT	Pelvic floor muscle training
PEDro	Physiotherapy Evidence Database
RUSI	Rehabilitative ultrasound imaging
