# Supplementary material for: Ultrasound-Based Biofeedback for Pelvic Floor Rehabilitation: A Systematic Review of Randomized Controlled Trials
Source: J Clin Med. 2026 Aug 19;15(16):6422. doi: 10.3390/jcm15166422 (PMC13514108; doi:10.3390/jcm15166422)
Supplement: Supplementary file 1 [file jcm-15-06422-s001.zip › jcm-4415614-checklist.pdf]

PRISMA 2020 Checklist

Ultrasound-Based Biofeedback for Pelvic Floor Rehabilitation: A Systematic Review of Randomized Controlled Trials

Section and topic	Item #	Checklist item	Location where item is reported
**TITLE**	1	Identify the report as a systematic review.	Title, p. 1
**ABSTRACT**	2	See the PRISMA 2020 for Abstracts checklist.	Abstract, p. 2
**INTRODUCTION — Rationale**	3	Describe the rationale for the review in the context of existing knowledge.	Introduction, pp. 2–3
**INTRODUCTION — Objectives**	4	Provide an explicit statement of the objective(s) or question(s) the review addresses.	Introduction, p. 3
**METHODS — Eligibility criteria**	5	Specify the inclusion and exclusion criteria for the review and how studies were grouped for the syntheses.	Section 2.2, p. 4
**METHODS — Information sources**	6	Specify all databases, registers, websites, organisations, reference lists and other sources searched or consulted to identify studies. Specify the date when each source was last searched or consulted.	Sections 2.1 and Table 1, pp. 3–5
**METHODS — Search strategy**	7	Present the full search strategies for all databases, registers and websites, including any filters and limits used.	Table 1, p. 5
**METHODS — Selection process**	8	Specify the methods used to decide whether a study met the inclusion criteria, including how many reviewers screened each record and report, whether they worked independently, and how disagreements were resolved.	Section 2.2, p. 4
**METHODS — Data collection process**	9	Specify the methods used to collect data from reports, including how many reviewers collected data, whether they worked independently, processes for obtaining or confirming data, and how disagreements were resolved.	Section 2.3, p. 4
**METHODS — Data items: outcomes**	10a	List and define all outcomes for which data were sought. Specify whether all compatible results were sought and, if not, the methods used to decide which results to collect.	Section 2.3, p. 4
**METHODS — Data items: other variables**	10b	List and define all other variables for which data were sought. Describe assumptions made about missing or unclear information.	Section 2.3, p. 4
**METHODS — Study risk of bias assessment**	11	Specify the methods used to assess risk of bias in the included studies, including details of the tool(s), how many reviewers assessed each study and whether they worked independently.	Section 2.4, p. 6
**METHODS — Effect measures**	12	Specify for each outcome the effect measure(s) used in the synthesis or presentation of results.	Not explicitly prespecified; numerical measures reported in Table 4, pp. 9–10
**METHODS — Synthesis methods: eligibility**	13a	Describe the processes used to decide which studies were eligible for each synthesis.	Sections 2.2–2.3, p. 4
**METHODS — Synthesis methods: data preparation**	13b	Describe any methods required to prepare the data for presentation or synthesis, such as handling missing summary statistics or data conversions.	Not required / not reported
**METHODS — Synthesis methods: tabulation and visual display**	13c	Describe any methods used to tabulate or visually display results of individual studies and syntheses.	Section 2.3 and Tables 2–4, pp. 4, 7–10
**METHODS — Synthesis methods: synthesis model**	13d	Describe any methods used to synthesize results and provide a rationale. If meta-analysis was performed, describe the model, heterogeneity methods and software.	Narrative synthesis; meta-analysis not performed because of clinical and methodological heterogeneity, pp. 12–13
**METHODS — Synthesis methods: heterogeneity exploration**	13e	Describe any methods used to explore possible causes of heterogeneity among study results.	Not applicable; only two clinically heterogeneous studies
**METHODS — Synthesis methods: sensitivity analyses**	13f	Describe any sensitivity analyses conducted to assess robustness of the synthesized results.	Not applicable
**METHODS — Reporting bias assessment**	14	Describe any methods used to assess risk of bias due to missing results in a synthesis.	Not formally assessed because too few studies; pp. 12–13
**METHODS — Certainty assessment**	15	Describe any methods used to assess certainty (or confidence) in the body of evidence for an outcome.	Section 2.4, p. 6; Supplementary Table S1
**RESULTS — Study selection**	16a	Describe the results of the search and selection process, from the number of records identified to the number of studies included, ideally using a flow diagram.	Results and Figure 1, p. 6
**RESULTS — Excluded studies**	16b	Cite studies that might appear to meet the inclusion criteria but were excluded, and explain why they were excluded.	Reasons are summarized in Figure 1 and Results, p. 6; individual excluded reports are not listed
**RESULTS — Study characteristics**	17	Cite each included study and present its characteristics.	Sections 3.2–3.3 and Table 4, pp. 7–10
**RESULTS — Risk of bias in studies**	18	Present assessments of risk of bias for each included study.	Section 3.1 and Table 3, p. 7
**RESULTS — Results of individual studies**	19	For all outcomes, present summary statistics for each group and an effect estimate with its precision where available.	Section 3.4 and Table 4, pp. 8–10
**RESULTS — Results of syntheses: contributing studies**	20a	For each synthesis, briefly summarise the characteristics and risk of bias among contributing studies.	Sections 3.1–3.4, pp. 7–10

Section and topic	Item #	Checklist item	Location where item is reported
**RESULTS — Results of syntheses: statistical synthesis**	20b	Present results of all statistical syntheses conducted. If meta-analysis was done, present the summary estimate, precision and heterogeneity.	Not applicable; no meta-analysis
**RESULTS — Results of syntheses: heterogeneity investigations**	20c	Present results of investigations of possible causes of heterogeneity among study results.	Not applicable
**RESULTS — Results of syntheses: sensitivity analyses**	20d	Present results of all sensitivity analyses conducted.	Not applicable
**RESULTS — Reporting biases**	21	Present assessments of risk of bias due to missing results for each synthesis assessed.	Not formally assessable; pp. 12–13
**RESULTS — Certainty of evidence**	22	Present assessments of certainty (or confidence) in the body of evidence for each outcome assessed.	Section 3.1, p. 7; Supplementary Table S1
**DISCUSSION — Interpretation**	23a	Provide a general interpretation of the results in the context of other evidence.	Discussion, pp. 11–12
**DISCUSSION — Limitations of evidence**	23b	Discuss limitations of the evidence included in the review.	Discussion, pp. 12–13
**DISCUSSION — Limitations of review processes**	23c	Discuss limitations of the review processes used.	Discussion, pp. 12–13
**DISCUSSION — Implications**	23d	Discuss implications of the results for practice, policy and future research.	Discussion and Conclusions, p. 13
**OTHER INFORMATION — Registration and protocol**	24a	Provide registration information for the review, including register name and registration number, or state that the review was not registered.	PROSPERO CRD420261278982, Abstract p. 2 and Section 2.1, p. 4
**OTHER INFORMATION — Protocol access**	24b	Indicate where the review protocol can be accessed, or state that a protocol was not prepared.	PROSPERO record CRD420261278982, Section 2.1, p. 4
**OTHER INFORMATION — Amendments**	24c	Describe and explain any amendments to information provided at registration or in the protocol.	Not reported
**OTHER INFORMATION — Support**	25	Describe sources of financial or non-financial support for the review and the role of funders or sponsors.	Funding statement, p. 13
**OTHER INFORMATION — Competing interests**	26	Declare any competing interests of review authors.	Conflicts of Interest, p. 14
**OTHER INFORMATION — Availability of data, code and materials**	27	Report which materials are publicly available and where they can be found.	Data Availability Statement, p. 14; Supplementary Table S1
