# Supplementary material for: Ultrasound-Based Biofeedback for Pelvic Floor Rehabilitation: A Systematic Review of Randomized Controlled Trials
Source: J Clin Med. 2026 Aug 19;15(16):6422. doi: 10.3390/jcm15166422 (PMC13514108; doi:10.3390/jcm15166422)
Supplement: Supplementary file 1 [file jcm-15-06422-s001.zip › jcm-4415614-supplementary Table S1.pdf]

Supplementary Table S1. GRADE evidence profile

Ultrasound-based biofeedback for pelvic floor rehabilitation: certainty of evidence for outcomes reported in the two eligible randomized controlled trials.

Outcome	Study / population	N	Risk of bias	Inconsistency	Indirectness	Imprecision	Publication bias	Certainty	Main reasons for downgrading
Urinary leakage (24-h pad weight)	Galea et al. (2013) [24]; older women with urinary incontinence	22	Serious[1]	Not assessable[2]	Not serious	Very serious[3]	Not assessable[4]	**Very low** ⊕◯◯◯	High risk of bias, exploratory pilot design, very small sample, and no effect estimates or 95% CIs.
Weekly urinary incontinence episodes	Galea et al. (2013) [24]; older women with urinary incontinence	22	Serious[1]	Not assessable[2]	Not serious	Very serious[3]	Not assessable[4]	**Very low** ⊕◯◯◯	High risk of bias, small sample, and absence of precise between-group effect estimates.
Incontinence-related quality of life	Galea et al. (2013) [24]; older women with urinary incontinence	22	Serious[1]	Not assessable[2]	Not serious	Very serious[3]	Not assessable[4]	**Very low** ⊕◯◯◯	High risk of bias, small sample, single-study evidence, and no confidence intervals.
Pelvic pain	Kuo et al. (2024) [25]; postpartum women with pregnancy-related pelvic girdle pain	53	Serious[5]	Not assessable[2]	Serious[6]	Not serious[7]	Not assessable[4]	**Low** ⊕⊕◯◯	High risk of bias and indirectness because ultrasound was embedded within a multimodal programme; evidence from one trial.
Disability	Kuo et al. (2024) [25]; postpartum women with pregnancy-related pelvic girdle pain	53	Serious[5]	Not assessable[2]	Serious[6]	Not serious[7]	Not assessable[4]	**Low** ⊕⊕◯◯	High risk of bias and indirectness; superiority over exercise without biofeedback was not demonstrated.
Walking performance (6-m walk test)	Kuo et al. (2024) [25]; postpartum women with pregnancy-related pelvic girdle pain	53	Serious[5]	Not assessable[2]	Serious[6]	Not serious[7]	Not assessable[4]	**Low** ⊕⊕◯◯	High risk of bias, indirectness, and evidence from a single small trial.
Pelvic floor muscle contractility	Kuo et al. (2024) [25]; postpartum women with pregnancy-related pelvic girdle pain	53	Serious[5]	Not assessable[2]	Serious[6]	Serious[8]	Not assessable[4]	**Very low** ⊕◯◯◯	High risk of bias, indirectness, and wide confidence intervals including no effect.

Notes.

Certainty started at high because all included studies were randomized controlled trials. Certainty was downgraded according to the GRADE domains: risk of bias, inconsistency, indirectness, imprecision, and publication bias.

[1] Downgraded one level for risk of bias because Galea et al. excluded one randomized participant as an extreme outlier, did not use intention-to-treat analysis, and presented concerns regarding selective reporting.

[2] Inconsistency could not be formally assessed because each outcome was informed by a single study; no additional downgrade was applied.

[3] Downgraded two levels for very serious imprecision because Galea et al. was an exploratory pilot trial with a very small sample and did not report effect estimates or 95% confidence intervals.

[4] Publication bias could not be formally assessed because fewer than ten studies were available; no downgrade was applied, although publication bias cannot be excluded.

[5] Downgraded one level for risk of bias because Kuo et al. had substantial losses to follow-up, used an available-case analysis, and presented concerns related to blinding and selective reporting.

[6] Downgraded one level for indirectness because ultrasound biofeedback was embedded within a multimodal pelvic floor and stabilization programme in a specific postpartum population.

[7] No downgrade for imprecision because comparisons with education showed clinically relevant effects with confidence intervals excluding no effect, although superiority over exercise without biofeedback was not consistently demonstrated.

[8] Downgraded one level for imprecision because confidence intervals were wide and included no effect for the comparison with exercise without biofeedback.

Abbreviations: BIO+EXE, integrated exercise plus ultrasound biofeedback; CI, confidence interval; GRADE, Grading of Recommendations Assessment, Development and Evaluation; N, number of participants; PFM, pelvic floor muscle; RCT, randomized controlled trial.
